# Supplementary material for: Efficacy of 4% articaine vs 2% lidocaine in mandibular and maxillary block and infiltration anaesthesia in patients with irreversible pulpitis: a systematic review and meta-analysis
Source: PeerJ. 2021 Sep 24;9:e12214. doi: 10.7717/peerj.12214 (PMC8475541; doi:10.7717/peerj.12214)
Supplement: Supplemental Information 2 [file peerj-09-12214-s002.docx]

**Systematic Review and Meta-Analysis Rationale:**

The purpose of the present systematic review and meta-analysis is to assess the efficacy of 4% Articaine versus 2% Lidocaine in the mandibular and maxillary block and infiltration anaesthesia in patients with irreversible pulpitis.

Highlights:

1. Successful pulpal anaesthesia is the cornerstone for painless root canal treatment, especially in patients with symptomatic pulpitis.
2. Articaine was introduced to overcome supplemental anaesthesia and to increase the effectiveness of the quality of anaesthesia.
3. Articaine is associated with a lower visual analogue scale rating for pain.
4. Articaine resulted in 1.37-fold and 1.06-fold higher clinical success rate than lidocaine for mandibular and maxillary teeth respectively.
